# An Unusual Presentation of Cutaneous Leishmaniasis in a Patient with Substance Use: A Diagnostic and Management Case Report

**DOI:** 10.1002/ccr3.72361

**Published:** 2026-04-27

**Authors:** Hossein Pazoki, Eissa Soleymani, Shirafkan Kordi, Mahdi Fakhar

**Affiliations:** ^1^ Department of Medical Microbiology, Faculty of Medicine, Infectious Diseases Research Center Gonabad University of Medical Science Gonabad Iran; ^2^ Toxoplasmosis Research Center, Communicable Diseases Institute Mazandaran University of Medical Sciences Sari Mazandaran Iran; ^3^ Department of Parasitology and Mycology, Faculty of Medicine Mazandaran University of Medical Sciences Sari Iran; ^4^ Department of Infectious Diseases, Antimicrobial Resistance Research Center, Faculty of Medicine Mazandaran University of Medical Sciences Sari Iran; ^5^ Gastroenterology and Hepatology Diseases Research Center, Interdisciplinary Research Committee, Shahid Beheshti Hospital Qom University of Medical Sciences Qom Iran

**Keywords:** addiction, atypical ulcers, cutaneous leishmaniasis, *leishmania tropica*, PCR

## Abstract

In patients with atypical ulcers and travel history to endemic areas, a comprehensive diagnostic workup for cutaneous leishmaniasis is imperative. This case confirms *Leishmania tropica* through combined microscopy, culture, and PCR, leading to successful treatment with systemic glucantime and adjunctive wound care.

## 1 Introduction

Leishmaniasis is classified as a parasitic infection that is recognized as one of the 20 neglected tropical diseases (NTDs) [1]. Cutaneous leishmaniasis (CL) is caused by protozoa of the genus *Leishmania* which are transmitted through the bite of an infected sandfly and are widespread in tropical and subtropical regions such as Iran [2]. WHO estimates that 600,000 to 1 million new cases of CL occur worldwide each year, and about 95% of CL cases are reported in the Americas, the Middle East, the Mediterranean area, and Central Asia [3].

It can be caused by several *Leishmania* spp., and is transferred to human beings and animals by sandflies, *Phlebotomus*, and *Lutzomyia* species. Clinical manifestations of CL have typical and atypical forms, and atypical forms have diverse clinical manifestations and can be confused with other skin diseases. The diverse clinical manifestations depend on both the infecting parasite species and the host's immune response, which makes their correct diagnosis challenging. Microscopic and molecular examination is critical in diagnosing CL [4, 5, 6]. Here we present an unusual case of a 48‐year‐old drug addict who was admitted to Qaemshahr Hospital with large eczematous ulcers on both his right foot and hand.

## 2 Case History/ Examination

**FIGURE 1:**
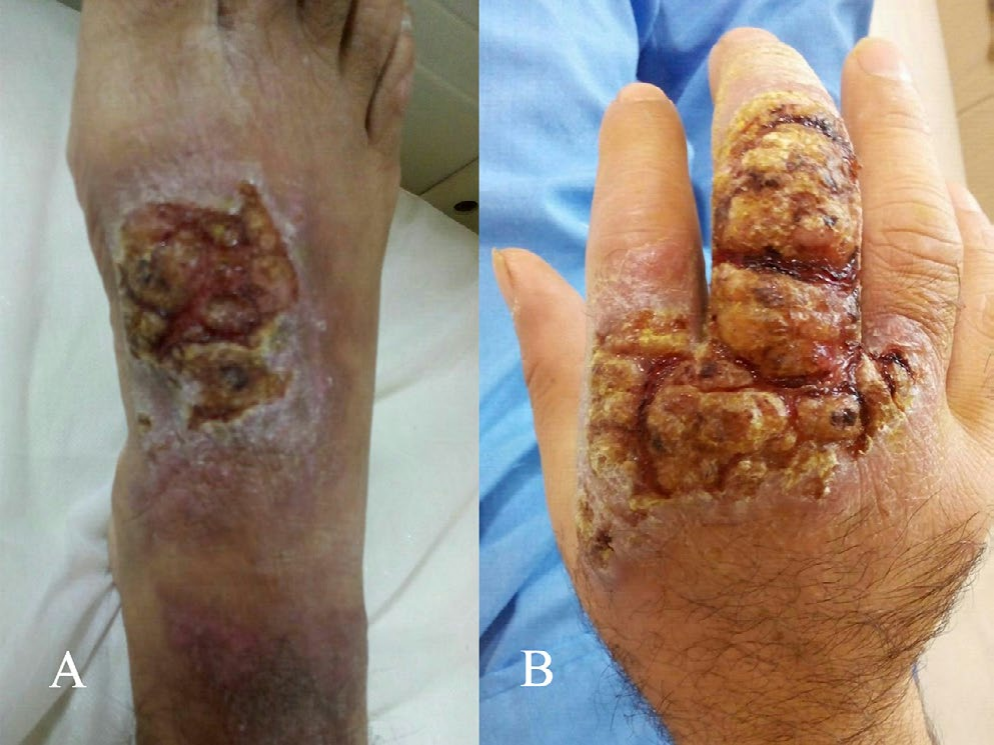
Large eczematous ulcers due to cutaneous leishmaniasis on the dorsal of the right foot (A) and hand (B) of the patient before treatment.

A 48‐year‐old man with a history of addiction was admitted to the infectious ward of Razi Teaching Hospital in Qaemshahr town, Mazandaran Province, northern Iran. Sixteen months ago, he had traveled to Razavi Khorasan province, an endemic area for CL disease. He presented with large, painful, and ulcerated lesions on his right hand and foot (Figure 1A,B). Pus and malodorous fluids were extracted from the lesions. The patient reported that his pain subsided after using opium. The patient had a long‐standing history of opium dependence (smoked form) for several years and reported regular use, particularly to alleviate pain associated with the lesions. There was no history of intravenous drug use. During hospitalization, the patient initially experienced mild withdrawal symptoms, including anxiety and insomnia, on the third day of admission. He was evaluated by the hospital's internal medicine team, and a consultation with the psychiatric service was offered; however, the patient declined formal psychiatric assessment and addiction counseling. Withdrawal symptoms were managed supportively without pharmacological intervention, as the patient refused replacement therapy. Harm reduction education was provided by the nursing staff regarding the risks of substance use on wound healing and immune function. The patient continued to use opium intermittently during hospitalization, which he reported as self‐medication for pain, though this was discouraged by the medical team. Pain management was subsequently optimized using non‐opioid analgesics to reduce his reliance on opium.

## 3 Methods (Differential Diagnosis, Investigations and Treatment)

Based on clinical manifestations and travel history to an endemic area, which were suggestive of CL, the patient was referred to the Regional Leishmaniasis Reference Laboratory (RLRL) at Mazandaran University of Medical Sciences for confirmatory testing. A comprehensive parasitological workup was performed, including direct smear microscopy of the lesions and culture in both Neal, Novy, Nicolle (NNN) medium and Roswell Park Memorial Institute (RPMI) 1640 medium (Gibco, Thermo Fisher Scientific, MA, USA).

Molecular analysis was also conducted. Consequently, a dermal scraping specimen was obtained from the patient's right hand and foot. A direct smear was prepared and stained with Giemsa stain, the gold standard procedure. Examination under a light microscope revealed the presence of intracellular and extracellular amastigotes (known as Leishman bodies). Upon examination of the smear, numerous amastigotes were identified. Promastigotes emerged from the amastigotes after three days in the NNN culture medium. In the RPMI culture medium, promastigotes were observed five days later. Additionally, a molecular method was employed to determine the presence of *Leishmania* species. DNA extraction was conducted by scraping samples from each slide with a clean blade, followed by a modified salting‐out procedure for DNA purification. A conventional PCR was utilized to identify the *Leishmania* spp. The PCR assay targeted the conserved region of *Leishmania* kinetoplast minicircles using the species‐specific primers LINR4 (forward, 5′‐GGG GTT GGT GTA AAA TAG GG‐3′) and LIN17 (reverse, 5′‐TTT GAA CGG GAT TTC TG‐3′), as described by Karamian et al. [2]. These primers amplify the variable region of the kinetoplast DNA (kDNA), yielding products of species‐specific lengths: approximately 650 base pairs (bp) for *L. major* and 720 bp for *L. tropica* [2].

After PCR analysis with specific primers, it was determined that *Leishmania tropica* was the primary cause of the lesion.

## 4 Conclusions and Results (Outcome and Follow‐Up)

**FIGURE 2:**
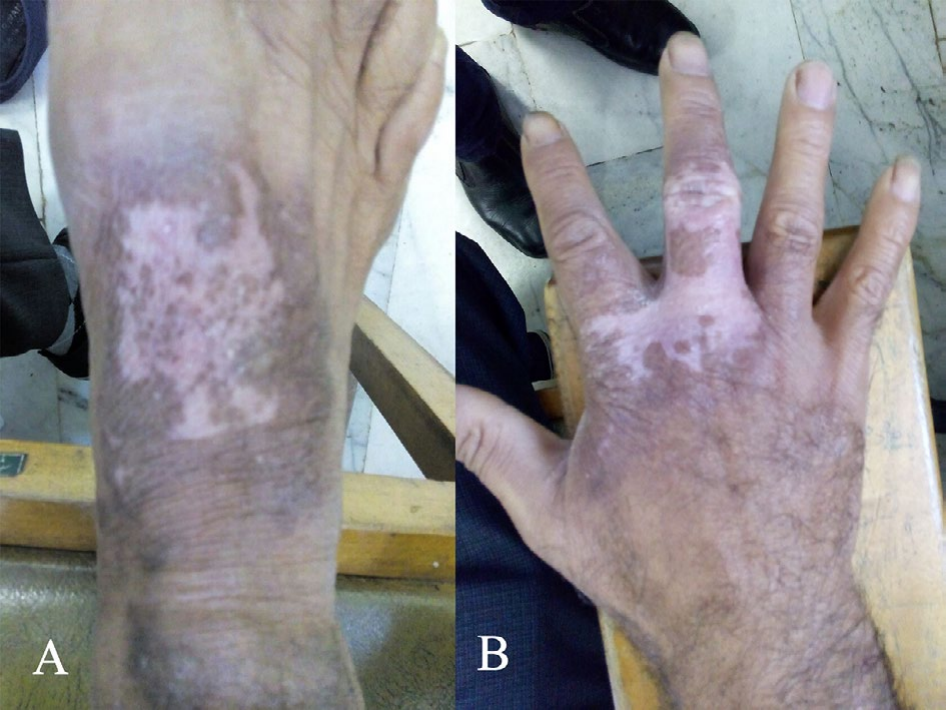
Wounds healing post suitable treatment in patient with cutaneous leishmaniasis due to Leishmania tropica on the dorsal of right foot (A) and hand (B).

The patient achieved full recovery following systemic treatment with meglumine antimoniate (Glucantime, France), in a dose of 20 mg/kg/day for 28 days, leaving only one scar with color change at the site of the previous lesion. In addition, to address secondary infection and promote wound healing, 2% Mupirocin topical ointment was applied three times daily for 15 days, followed by 25% Zinc oxide cream twice daily until complete resolution of symptoms (Figure 2A,B). Furthermore, during a follow‐up examination approximately one year later, no recurrence of the lesion was observed.

## 5 Discussion

CL is the most common form of leishmaniasis, endemic in 87 countries and affecting millions of people worldwide [3]. CL primarily impacts populations facing poverty, low levels of public health, malnutrition, weakened immune systems, poor housing, and population displacement [7]. This article presents an unusual case of CL, emphasizing its clinical presentation, diagnostic challenges, treatment, and public health implications. The gold standard for diagnosing cutaneous leishmaniasis is parasitological diagnosis, known for its high specificity. This method involves microscopic examination using Giemsa‐stained biopsy smears or aspirates, culture of biopsy triturates or aspirates, or histopathological testing of fixed lesion biopsies [8, 9]. Microscopic examination is the most common diagnostic method used due to the limited availability and high cost of more advanced techniques at tertiary healthcare levels in endemic areas. However, this method does not identify the specific species of CL. One of the most effective parasitological diagnoses for CL is molecular methods, primarily utilizing PCR techniques, especially in cases with low parasite load [2, 9].

A crucial aspect of diagnosing the disease is identifying the patient's history, including their residence in a non‐endemic region and any travel to endemic areas [10]. This information can play a significant role in diagnosing and treating the disease early. For instance, if a patient residing in a non‐endemic region like northern Iran presents with symptoms of leishmaniasis, the awareness of the possibility of the disease can lead to prompt diagnosis and treatment, thereby preventing further spread within the community. Additionally, infections occurring in individuals living in non‐endemic areas may manifest as more severe forms of CL [11, 12].

The classic clinical presentation of CL typically begins as a painless erythematous papule at the site of a sandfly bite, which gradually enlarges and may evolve into a well‐demarcated ulcer with raised, indurated borders and a central crater commonly described as a “volcano‐like” lesion. In many cases, the ulcer has a dry base and limited surrounding inflammation [8, 11].

In contrast, our patient presented with unusually large, painful, and exudative lesions with an eczematous appearance, ill‐defined margins, and purulent discharge. The presence of malodorous secretions and extensive inflammation suggested secondary bacterial infection and obscured the typical morphology of CL. This deviation from the classic volcano‐like ulcer, together with the lesion size and chronicity, supports the characterization of this case as an atypical presentation.

Opium consumption can indeed have detrimental effects on the healing process of *Leishmania* wounds. One significant impact is the suppression of the immune system, making it harder for the body to combat infections, including those caused by the *Leishmania* parasite. This can prolong the healing time and increase the risk of complications [13, 14]. Proper wound care is crucial in the treatment of CL. Alongside drug therapy, effective wound care measures like debridement and appropriate dressing play a key role in the cure of CL. However, opium use can impede the level of wound care received by individuals. The effects of opium, such as blurred vision, drowsiness, and overall neglect of health, can complicate the healing process and delay recovery [14, 15]. In this patient's case, opium addiction and use as a pain reliever may have contributed to the spread of the CL ulcer. It's essential to address substance abuse issues alongside the treatment of CL to promote successful wound healing and overall recovery.

The systemic treatment with glucantime proved successful in the case, suggesting its recommendation for similar instances where lesions are large in size or number. Additionally, the use of ointments, antibacterial, and antifungal medications in CL wounds with secondary infections significantly impacts wound healing. Among these, mupirocin ointment stands out as a broad‐spectrum antibiotic effective in treating bacterial skin infections. It plays a crucial role in eliminating secondary bacterial infections, as observed in our patient [16, 17]. Another beneficial option is zinc oxide ointment, known for its wound‐healing properties in the treatment of leishmaniasis. Research has shown that zinc oxide promotes wound healing, reduces inflammation, and enhances re‐epithelial production. When used in conjunction with other medications, it can help lower the incidence of skin infections [18].

CL is an endemic disease in Iran, with rare reports in Mazandaran province. However, the high frequency of travel outside the province raises concerns about the potential spread of CL to non‐endemic areas. Furthermore, in non‐endemic regions, the presence of atypical cases of CL accompanied by secondary bacterial infections can complicate the diagnosis of CL. In such scenarios, it is advisable to utilize culture and molecular methods for accurate diagnosis.

While optimal management of CL in patients with substance use disorder ideally includes integrated addiction treatment, our patient's refusal of psychiatric consultation and formal counseling presented a significant challenge. This highlights a common clinical dilemma in resource‐limited settings where patients may not accept comprehensive care. In such cases, harm reduction strategies including education on the negative impact of substance use on wound healing and provision of alternative pain management become essential components of care. The successful treatment outcome in this patient, despite ongoing intermittent opium use, may be attributed to the combination of systemic antimonial therapy, aggressive management of secondary bacterial infection, and optimized wound care. Nevertheless, this case underscores the importance of addressing substance use disorders in CL management and the need for patient‐centered approaches that respect autonomy while promoting recovery.

CL infections in individuals residing in non‐endemic regions may present as more severe forms of the disease. In addition to treating CL, it is crucial to address any substance abuse issues in patients. This holistic approach is necessary to facilitate successful wound healing, prevent serious complications, and promote overall recovery. Integrating substance abuse treatment alongside CL management can improve patient outcomes and quality of life.

## Author Contributions

**Hossein Pazoki:** writing – original draft. **Eissa Soleymani:** investigation, project administration, writing – review and editing. **Shirafkan Kordi:** methodology, validation. **Mahdi Fakhar:** investigation, supervision, writing – review and editing.

## Funding

The authors have nothing to report.

## Ethics Statement

The study was approved by our local ethics committee.

## Consent

Written informed consent was obtained from the patient for publication of this report.

## Conflicts of Interest

The authors declare no conflicts of interest.

## Data Availability

The data are available with the correspondence author and can be reached on request.
